# Genetic Pool Information Reflects Highly Suitable Areas: The Case of Two Parapatric Endangered Species of Tuco-tucos (Rodentia: Ctenomiydae)

**DOI:** 10.1371/journal.pone.0097301

**Published:** 2014-05-12

**Authors:** Daniel Galiano, Jorge Bernardo-Silva, Thales R. O. de Freitas

**Affiliations:** 1 Programa de Pós-Graduação em Biologia Animal, Universidade Federal do Rio Grande do Sul, Porto Alegre, RS, Brazil; 2 Departamento de Genética, Universidade Federal do Rio Grande do Sul, Porto Alegre, RS, Brazil; Smithsonian Conservation Biology Institute, United States of America

## Abstract

Conservation of small mammals requires knowledge of the genetically and ecologically meaningful spatial scales at which species respond to habitat modifications. Conservation strategies can be improved through the use of ecological niche models and genetic data to classify areas of high environmental suitability. In this study, we applied a Maxent model integrated with genetic information (nucleotide diversity, haplotype diversity and Fu's *Fs* neutrality tests) to evaluate potential genetic pool populations with highly suitable areas for two parapatric endangered species of tuco-tucos (*Ctenomys minutus* and *C. lami*). Our results demonstrated that both species were largely influenced by vegetation and soil variables at a landscape scale and inhabit a highly specific niche. *Ctenomys minutus* was also influenced by the variable altitude; the species was associated with low altitudes (sea level). Our model of genetic data associated with environmental suitability indicate that the genetic pool data were associated with highly suitable areas for *C. minutus*. This pattern was not evident for *C. lami*, but this outcome could be a consequence of the restricted range of the species. The preservation of species requires not only detailed knowledge of their natural history and genetic structure but also information on the availability of suitable areas where species can survive, and such knowledge can aid significantly in conservation planning. This finding reinforces the use of these two techniques for planning conservation actions.

## Introduction

Subterranean rodents of the genus *Ctenomys* inhabit the southern part of the Neotropical region from the extreme south up to southern Peru, including the entire Patagonian region and showing a wide latitudinal variation. The range of the genus extends from sea level to 4,000 m in the Andean region [1]. These small subterranean herbivores are among the most geographically variable mammals and are also the most speciose of all subterranean rodent groups [2]. Presently, the genus includes approximately 60 species [2]. South American tuco-tucos (*Ctenomys*) have attracted special interest in terms of speciation and evolution by virtue of their patchy distributions, low vagility, territoriality, and extensive karyotypic variation [1].

In the coastal plain of southern Brazil, the species with the widest geographic distribution is *Ctenomys minutus*, which inhabits sandy fields and dunes and has a range extending from Jaguaruna Beach in the state of Santa Catarina to the town of São José do Norte in the state of Rio Grande do Sul. *Ctenomys lami* is an endemic species inhabiting a sandy region named ‘Coxilha das Lombas’ along a narrow line of old dunes that extends from north of Guaiba Lake to the northwestern sandbanks of Barros Lake [3], [4]. A hybrid zone between *C. minutus* and *C. lami* is also present. It is probable that this zone was formed due to habitat alterations. A wide humid zone once separated both species and represented a barrier [5]. *Ctenomys lami* is cited as vulnerable in the Red List of the International Union for Conservation of Nature [6], and *C. minutus* is listed as data deficient. However, given the current state of information about tuco-tucos in Brazil, Fernandes et al. [7] have affirmed that the vulnerability of these subterranean rodents is greater than presently supposed and that any conservation effort should be based on consistent and detailed studies of habitat occupation.

Ecological niche models (ENMs), based on the use of multiple strategies in conservation plans, are an important tool for determining the distribution of threatened species for conservation purposes [8]. In particular cases of restricted distribution or sparse populations of rare or endangered species, the use of ENMs may be necessary to identify a set of areas for protection [9]. Functionally, ENMs could be considered to furnish a specification of the relationship between the points of occurrence of a species and a set of multivariate environmental data [10]. The need to base strategies of ENM development on niche theory facilitates the interpretation and discussion of the resulting models relative to conservation issues. Conservation of small mammals requires knowledge of the genetically and ecologically meaningful spatial scales at which species respond to habitat modification. Understanding this information is important because small mammals represent a major assemblage of species occupying the most varied environments [11]. Large-scale, presence-only models are vital to complement the information obtained by local studies. They reveal otherwise-overlooked ecological requirements by identifying the environmental parameters that influence species distributions on a broad geographical scale [12].

*Ctenomys minutus* and *C. lami* inhabit the coastal plain of southern Brazil. Several studies have addressed the degradation of this coastal environment, identifying both natural and human-induced factors contributing to long- and short-term change [13], [14]. Anthropogenic changes, such as urbanization in active dune areas [14], [15], have modified the natural landscape over the years. As a result of human activity, native habitats have become increasingly fragmented or destroyed, changing the patterns of gene flow between populations and modifying the levels of genetic diversity. In our particular case, conservation strategies can be improved with the use of ENMs and genetic data to classify areas of high environmental suitability and to investigate how genetic characteristics diverge in different areas.

In this study, we applied ENMs integrated with genetic information to evaluate potential genetic pool populations with highly suitable potential areas. We developed maximum-entropy (Maxent, Phillips et al. [10]) presence-only distribution models for *C. lami* and *C. minutus* based on the following goals:

1. To conduct the first geographical distribution analysis for both species over their entire distributional range;

2. To determine which ecological factors may be limiting the distributions of the species;

3. To develop an integrated framework to evaluate geographic patterns of genetic diversity within potential genetic pools;

4. To discuss conservation issues in the light of this concatenated approach.

## Methods

### Study area

**Figure 1:**
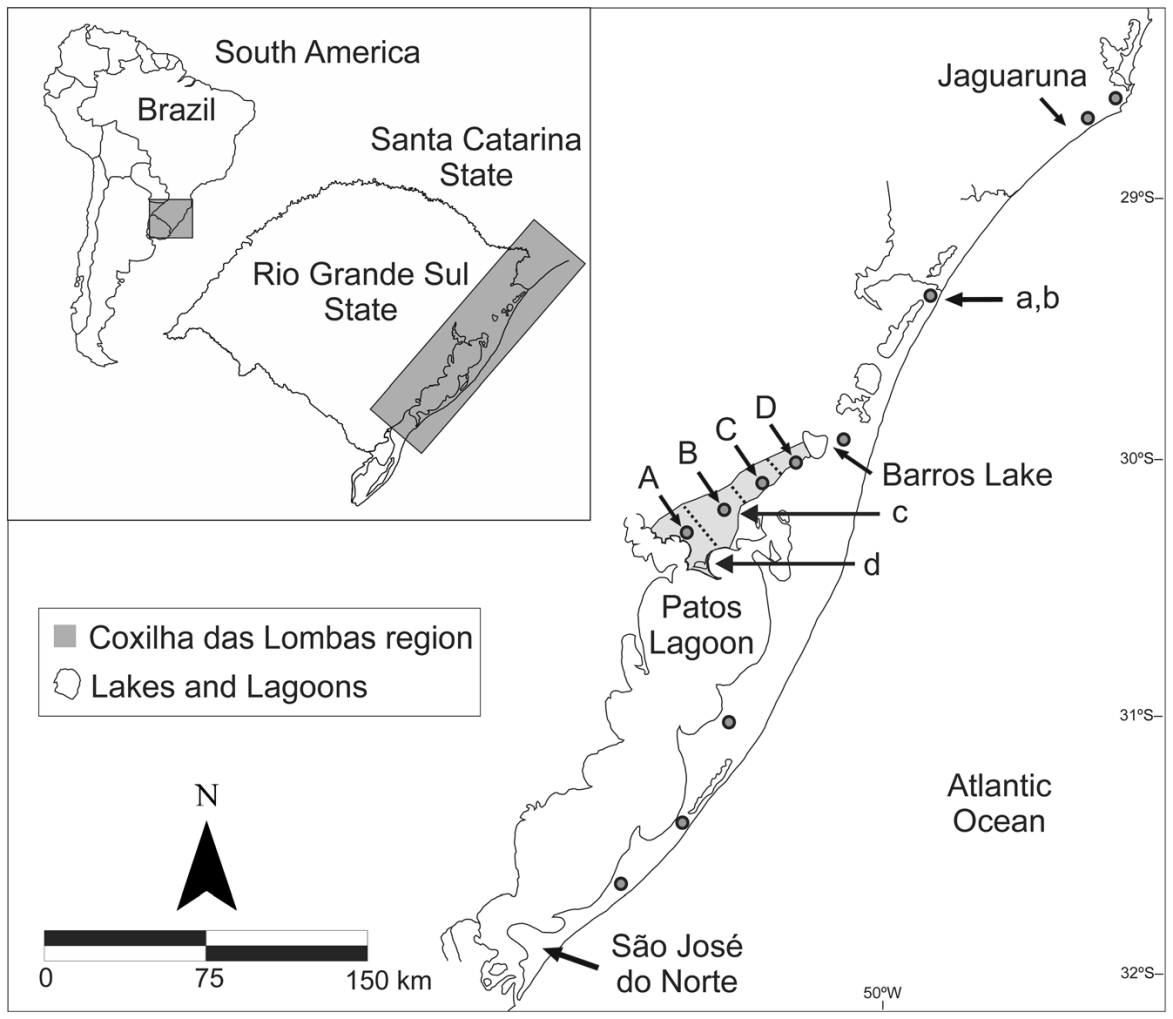
Coastal plain of southern Brazil with geographic details. The most important lakes and rivers are shown. Points (•) represent geographical regions for which genetic data are available from Lopes and Freitas (2012) and Lopes et al. (2013). In the region of Coxilha das Lombas are displayed the four karyotypic blocks described by Freitas (2007) (A, B, C and D). Conservation areas within the distribution of each species are also displayed (a, b: Parque de Itapeva and Parque da Guarita; c: Parque de Itapuã; d: Refugio de Vida Silvestre Banhado dos Pachecos).

The study was conducted on the southern coast of Brazil. The environment consists of sand dunes and relict ‘restinga’ forests [16]. The landscape of the coastal plain of southern Brazil is characterized by lakes, lagoons, rivers, and dunes that represent natural geographical barriers for different populations of *Ctenomys* [4], [17] (Figure 1).

### Species records

Current knowledge about the distributions of *C. minutus* and *C. lami* is based on records from the collection of the Laboratório de Citogenética e Evolução of the Universidade Federal do Rio Grande do Sul and collected by researchers [3]–[5], [18]–[21]. All of the coordinates were recorded using a GPS at the exact point of collection or observation. We considered a total of 74 records for *C. minutus* (n = 45) and *C. lami* (n = 29), each representing an established population of these tuco-tucos, to generate the ENMs (Figure S1).

### Environmental data

We used 19 Worldclim bioclimatic variables obtained through interpolated data from derived rainfall and temperature, with a resolution of 2.5 arc-minutes (five kilometers); one altitude variable [22], available at http://www.worldclim.org/download; and two categorical variables for soil and vegetation composition [23] available at http://mapas.mma.gov.br/i3geo/datadownload.htm. We included those two categorical variables due to their recognized ecological importance to the study species and genus [24]–[27]. The vegetation and soil maps were converted to rasters at the same level of resolution as the bioclimatic variables using ArcMap v. 10.0. We generated a matrix with the values of each climatic variable for the entire study area. We performed a Principal Components Analysis (PCA) on this matrix to identify correlations between variables, selecting the axes that explained ≥95% of the correlation structure. From this result, we selected variables with the highest absolute coefficient in each axis. This procedure yielded seven variables for *C. minutus* (mean temperature of coldest quarter, precipitation seasonality, mean temperature of warmest quarter, mean diurnal range (mean of monthly (max temp - min temp)), soil, vegetation, and altitude) and five variables for *C. lami* (maximum temperature of warmest month, minimum temperature of coldest month, mean temperature of warmest quarter, soil, and vegetation). To avoid information loss, we included all variables represented in the PCAs from both species to generate models for the two species. The models were constructed with a total of nine variables because equal variables were selected for the two species.

### Modeling

The models were constructed with Maxent version 3.3.3k [10], available at (http://www.cs.princeton.edu/~schapire/maxent/). Maxent estimates the ecological niche of a species by determining the distribution of maximum entropy (ME), subject to the constraint that the expected value of each environmental variable under this estimated distribution matches its empirical average. The technique was designed as a machine learning algorithm [10], [28], [29]. The default parameters in Maxent were used to construct the models: automatic features election, regularization multiplier at unity, maximum iterations 500 and convergence threshold 10^−5^. We produced maps of the potential distribution of the species using the logistic output format [29]. This format is an attempt to ensure the closest possible approach to an estimate of the probability that the species is present given the environment [28]. We chose Maxent because it requires only presence data, because it can process categorical data and model interactions, and because it has performed favorably when compared with alternative approaches [30], [31]. The models were validated by calculating the area under the curve (AUC) from a receiver operating characteristic curve (ROC). The relative importance of the variables was assessed with Maxent's built-in Jackknife functionality.

### Genetic data

Our basic data consisted of mitochondrial DNA (mtDNA) sequences corresponding to the cytochrome oxidase I gene and the hypervariable control region. The data were obtained from Lopes et al. [20] for *C. minutus* and Lopes and Freitas [19] for *C. lami*, where a detailed description of the genetic data can be obtained. For *C. minutus*, a total of 30 localities were sampled across the entire distribution of the species, and seven principal clades were highlighted in the Bayesian phylogenetic tree. We focused on these seven main genetic haplogroups described by Lopes et al. [20] (North 1, North 2, Coast, Barros Lake, Mostardas, Tavares, and South; see Lopes et al. [20] for details). For *C. lami*, 28 locations were sampled, and the data are presented by the authors based on the four karyotypic blocks proposed for the species (Block A, B, C and D; see Freitas [4], Lopes and Freitas [19]). We chose to present our results for this concatenated approach (genetic data and modeling) based on the haplogroups for *C. minutus* and karyotipic blocks for *C. lami* since we did not directly compare these two different units, but discuss the results independently for each species. We believe that the use of this approach may not overestimate the interpretation of the results.

Briefly, the geographical genetic structure of *C. minutus* was characterized by examining 340 individuals over the entire distributional range and using information from cytochrome oxidase I gene and control region sequences. For *C. lami*, a total of 178 specimens were sampled, using information from the same mitochondrial DNA markers. Our analyses were based primarily on three different genetic parameters obtained from genetic data estimated by these authors [19], [20]. The first parameter, nucleotide diversity (π), is defined by the equation
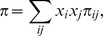
where *π_ij_* is the proportion of nucleotide differences between the *ith* and *jth* types of DNA sequences and *x_i_* and *x_j_* are the respective frequencies of these sequences. Nucleotide diversity is a measure of genetic variation. The second parameter, haplotype diversity (H_d_), is a measure of the uniqueness of a particular haplotype in a given population. H_d_ is defined by the equation
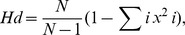
where *x_i_* is the (relative) haplotype frequency of each haplotype in the sample and N is the sample size. Haplotype diversity is given for each sample. The third parameter, Fu's *Fs*, is the probability of observing a random sample with a number of alleles equal to or smaller than the observed value given the observed level of diversity and the assumption that all of the alleles are selectively neutral. If we call this probability *Ŝ*, then
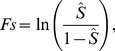
so that a negative value of *Fs* would be expected from a recent population expansion or from genetic hitchhiking. A positive value of *Fs* is evidence for a deficiency of alleles, as would be expected from a recent population bottleneck or from overdominant selection [32]. To display the genetic information on a map, we established a geographic coordinate associated with each haplogroup for *C. minutus* and with each karyotypic block for *C. lami*.

## Results

### The models

**Figure 2:**
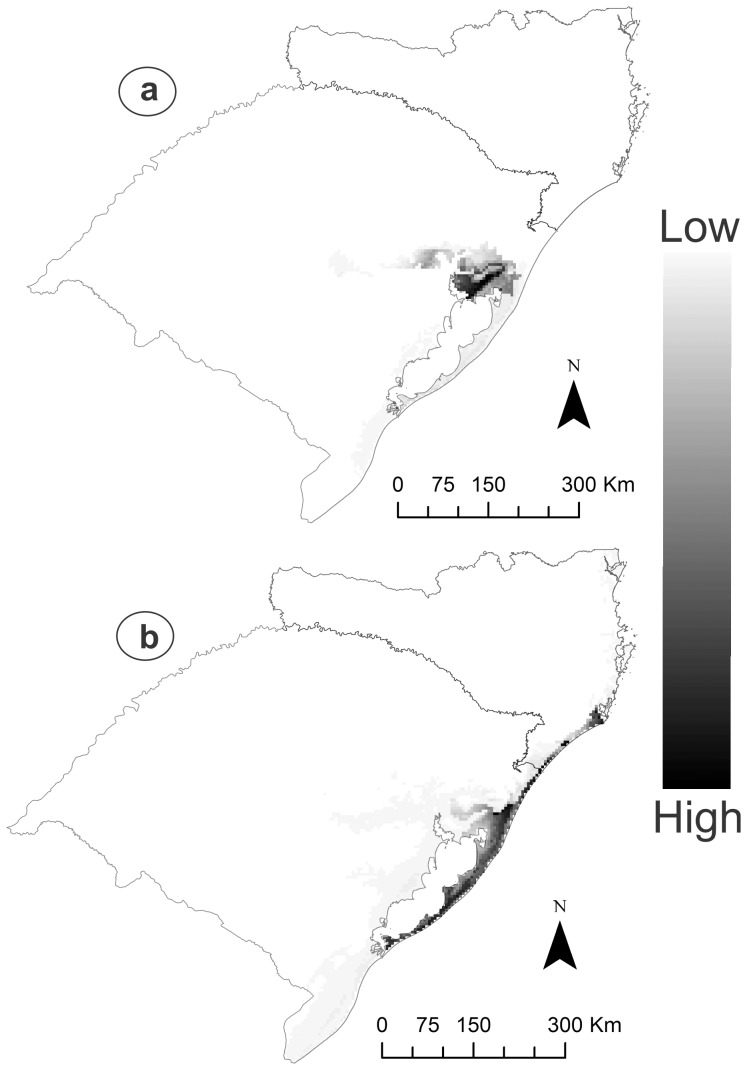
Suitable areas for (a) *Ctenomys lami* and (b) *Ctenomys minutus*, according to Maxent (maximum entropy) model in southern Brazilian coastal plain. Darker regions indicate greater ecological suitability.

The models constructed with Maxent produced distributional predictions for each species. The map with the widest distributional area was generated for *C. minutus*, whereas *C. lami* presented a very narrow distribution probability (Figure 2).

**Figure 3:**
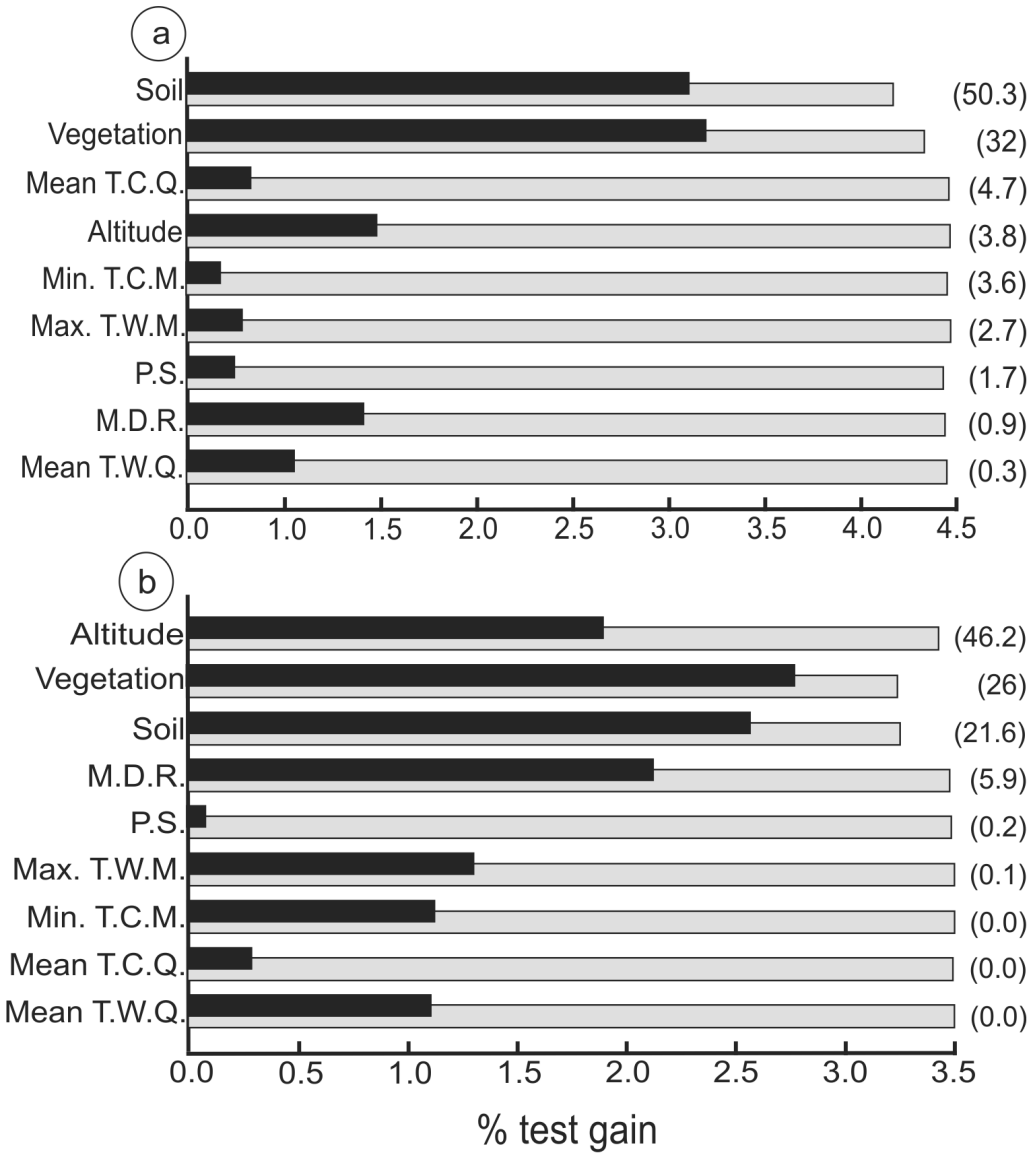
Jackknife analyses of the importance of environmental variables in maximum entropy modeling of *C. lami* (a) and *C. minutus* (b) occurrence. A heuristic estimate of the relative contribution of each variable to the global model is given in parentheses, with variables listed in descending order of importance. Grey bars show the performance of the global model (known as test gain) without each variable, and black bars show the influence of each variable in isolation (derived from a univariate model only). The variables are the following: Mean T.C.Q.: mean temperature of coldest quarter; Min. T.C.M.: minimum temperature of coldest month; Max. T.W.M.: maximum temperature of warmest month; P.S.: precipitation seasonality; M.D.R.: mean diurnal range (mean of monthly (max temp - min temp)); Mean T.W.Q.: mean temperature of warmest quarter.

According to the ME model, the occurrence of *C. lami* was most strongly associated with soil and vegetation. The variable with the highest gain when used in isolation was vegetation. For this reason, vegetation appears to be the single variable that furnishes the most useful information. The variable that decreased the gain most markedly when omitted was soil. For this reason, soil appears to furnish the greatest amount of information not present in the other variables. For *C. minutus*, occurrence was strongly associated with altitude, vegetation, and soil. The variable with highest gain when used in isolation was vegetation, and the variable that decreased the gain most markedly was also vegetation (Figure 3).

Both species were strongly influenced by vegetation and soil variables. Model performance, defined as the area under the curve, was highly discriminative for both species (AUC values for *C. lami* = 0.997 and *C. minutus* = 0.993), indicating that they inhabit a highly specific landscape niche.

### Genetic pool data

**Figure 4:**
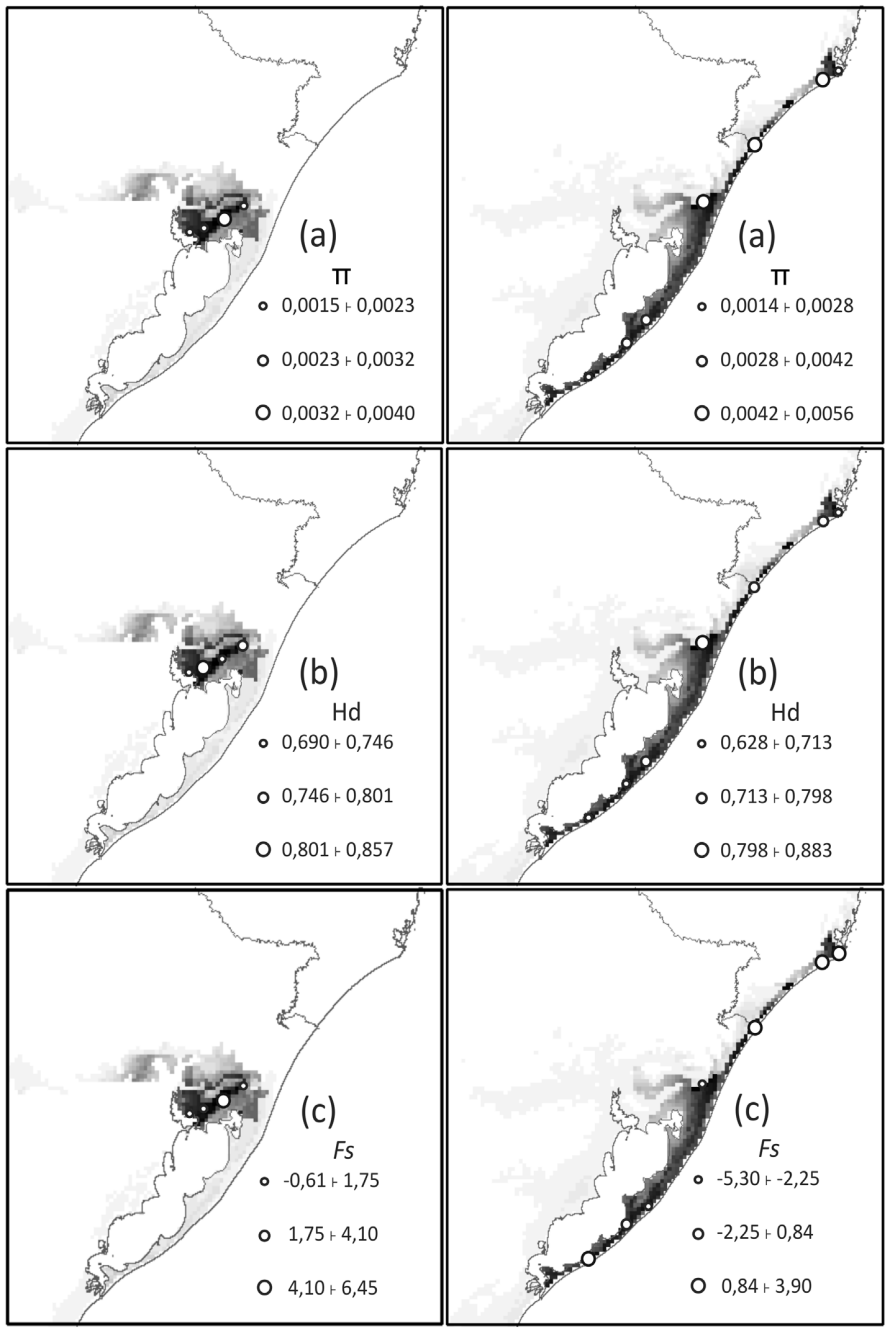
Geographical patterns of genetic parameters estimated within phylogeographic groups (*C. minutus*) and karyotype blocks (*C. lami*). Nucleotide diversity, *Π* (a); Haplotype diversity, *Hd* (b); Fu's neutrality parameter, *Fs* (c), overlapping the potential distribution of *C. lami* (left column) and *C. minutus* (right column). Darker regions indicate greater ecological suitability.

The three local genetic parameters estimated showed no apparent patterns for *C. lami*. The suitability values calculated between the blocks were the same (high), and block C showed the highest value of π; however, the same pattern was not followed by the H_d_ values, which were higher in blocks B and D. The *Fs* values were not significant for any of the analyzed blocks (Figure 4).

For *Ctenomys minutus*, areas of high suitability were in accordance with the seven principal genetic haplogroups found for the species (see Lopes et al. [20] for details). For π values, the northern portion of the distribution presented higher values (except for North 1). H_d_ presented the same trend, with the center of the distribution showing greater diversity. For the *Fs* index, only the Barros Lake haplogroup showed a significant result (−5.30), as would be expected from a recent population expansion or from genetic hitchhiking (Figure 4).

## Discussion

### Maximum entropy models and environmental variables

This is the first study to use ENM methods to test parameters that influence the occurrence of the genus *Ctenomys* and one of the first to use these techniques on subterranean mammals. The models presented here were successful in identifying regions that are suitable for the species. Both models showed few commission errors, such as the areas of medium environmental suitability on the east side of the Coxilha das Lombas region for *C. lami* and the areas of low suitability in the countryside along Patos Lagoon for *C. minutus*. In addition, model performance was highly discriminative for both species. Although we identified these commission errors, we must consider that is very difficult to incorporate the dispersal abilities of the species and geographical accessibility to the model (landscape configuration) at present or through history; however, this factor is necessary for the actual presence of species [33]. In our particular case, geographical accessibility is a very important issue because the tuco-tucos are characterized by limited individual mobility and a patchy distribution of local populations [25], [34]. *Ctenomys lami* appears to be restricted to the region of Coxilha das Lombas and showed a potential distribution at low to medium levels of occurrence beyond the limits of this region. The species was shown to be associated with local vegetation and soil type and to be geographically limited by the presence of wetlands and geographical inaccessibility, factors that characterize this region.

The distribution of *Ctenomys minutus* was restricted to the coastal plain of southern Brazil, including the region's first line of dunes and extending to sandy fields. This species shows a sympatric zone with another tuco-tuco species (*C. flamarioni*) in a range of approximately 15 km [35]. In an area extending to the northern portion of this region, *C. minutus* inhabits the first line of dunes. Below the sympatric zone, *C. minutus* occupies only the regions of sandy fields, although the ME model indicates that it can potentially occur up to the line of dunes. The reason for this apparent discrepancy is that near the southern limits of this sympatric zone, the first line of dunes is already occupied by *C. flamarioni* up to the southern boundary of Rio Grande do Sul state. This finding is, most likely, related to the historical patterns of occupation by these species. *Ctenomys minutus* shows a pattern of north-south occupation extending from Santa Catarina to Rio Grande do Sul state [20], and *C. flamarioni* shows a pattern of occupancy from south to north [36], [37]. This transition between the sand fields and dune line represents an environmental discontinuity along the range of *C. minutus*. According to Fornel et al. [38], who have found significant differences in skull morphology between specimens of *C. minutus* that inhabit sand fields and those that inhabit dunes, environmental characteristics such as soil hardness produce morphological adaptations in tuco-tucos. These authors found some variation among different populations belonging to different habitats in cranial shape, and these intraspecific phenotypic differences appear to arise as a combination of selection and drift acting as diversifying forces. However, according to our model of environmental suitability, this discontinuity may not inhibit free dispersal by specimens from different habitats. Therefore, these different environments may also be associated with distinct levels of environmental suitability, although we could not measure this difference in our models.

Although they are morphologically similar, both species studied here occupy distinct areas of the coastal plain. According to the ME models, *C. minutus* appears to show higher environmental plasticity than *C. lami*. The models proposed for these two species showed a low overlap of areas of high suitability. This characteristic appears to be defined primarily by the presence of the Coxilha das Lombas region and the geographical inaccessibility of this region. A hybrid zone between *C. lami* and *C. minutus* is present in the northern portion of this coxilha. This zone has been the subject of previous study. According to Gava and Freitas [5], it was formed by modifications of the environment because a humid zone that separated these species was drained for rice cultivation. Environmentally, our results suggest a medium probability of occurrence for both species in the region where they hybridize.

Moreover, edaphic conditions can be very important for the definition of a species' fundamental niche [39], [40]. The occurrence of *C. lami* and *C. minutus* was strongly influenced by soil and vegetation. *C. minutus* was also influenced by altitude, as it was associated with low altitudes (sea level). According to Kubiak [35], *C. minutus* is associated with areas with higher vegetation and biomass, which strengthens the evidence for the importance of this variable to the species. Given that tuco-tucos are exclusively subterranean, the use of variables that are directly linked to the species niche demonstrated to be extremely important in building the models, regardless of soil and vegetation variables, might have suffered from their degree of generalization. It is recommended that the choice of predictors in ENMs should consider the ecological relevancy of the predictors to the target species [41]. Such considerations are extremely important to improve conservation actions.

### Genetic information and conservation actions

Our analyses of the genetic data associated with the ME model for *C. lami* showed that there are differences in the levels of genetic diversity (π and H_d_) between populations, although the suitability is currently the same for all of the blocks. Lopes and Freitas [19] found that the genetic structure associated separately with each of the four different karyotypic blocks was inconsistent for mtDNA and microsatellite data. Environmentally, we also found no differences among these four karyotypic blocks. Thus, the presence of chromosomal rearrangements or distinct karyotypes between individuals noted by Freitas [4] does not appear to be limited by the environment.

Lopes et al. [20] recovered a pattern of genetic structure of the sampling sites subdivided into seven main haplogroups for *C. minutus*. In the current study, these haplogroups, represented by three genetic parameters, were associated with areas of high environmental suitability. This pattern may result from the occurrence of higher genetic diversity in the areas of higher suitability for the species. According to Lopes et al. [20], a relatively ancient genetic structure was present in the northern area of the geographical distribution, whereas the southern sampling sites may exhibit a founder effect of more recent occurrence. However, areas with high suitability showed different values of π and H_d_, thus other extrinsic (historical or contemporary) or intrinsic (behavioral) factors could be affecting the pattern of genetic diversity and structure.

The only value of the *Fs* index that was statistically significant for *C. minutus* was from the Barros Lake haplogroup, which was associated with a highly suitable area. As this pattern was not found for *C. lami* or for the other haplogroups of *C. minutus*, it is not possible to support our hypothesis that highly suitable areas favor population expansion or genetic hitchhiking, as indicated by a significant negative value of the *Fs* index.

The preservation of species requires not only detailed knowledge of their natural history and genetic structure but also information on the availability of suitable areas where species can survive; such knowledge can aid significantly in conservation planning. Both species analyzed here have suffered intense pressure from the fragmentation and reduction of their habitats. This process results in the death or isolation of individuals because these species are extremely niche-specific. The principal threats to *C. lami* are the progressive urbanization and human settlement in its territory, associated, for example, with agricultural activities [7], [19]. In the northern portion of the coast, *C. minutus* is threatened by land speculation associated with urban constructions along the coast and on the shores of the lagoon and by the fragmentation of the dune habitat. In the south, silviculture involving *Pinus* sp. directly affects the populations of the species ([7], authors' personal observations). A study by Bernardo-Silva et al. [42] focused on the conservation of two endangered species of red-bellied toads, and the authors suggested five important areas for species conservation based on a hotspot analysis. These areas included a significant number of threatened species, including *C. minutus*. Our results underscore that the areas proposed by those authors should be considered for reserve planning because they cover three highly suitable areas within the range of *C. minutus* (see Bernardo-Silva et al. [42]). Although their results did not focus on underground rodents, these hotspot areas are of great interest for *C. minutus* because considering conservation of habitats as a priority, the protection of sandy field areas with high suitability for underground tunneling is an interesting solution for protecting the species. Additionally, *C. minutus* has only two protected areas within its entire distribution (Parque de Itapeva and Parque da Guarita). These areas are in neighboring locations in the northeastern portion of the distribution. However, none of the southern populations are located in protected areas. Two state conservation units (Parque de Itapuã and Refugio de Vida Silvestre Banhado dos Pachecos) are located within the range of *C. lami*, but only parts of these areas overlap the distribution of the species. Over the past year, extensive field work has been conducted to search for *C. lami*, but few populations have been found outside these two units (authors' personal observations).

Our models of genetic data associated with environmental suitability indicated that genetic pool data were associated with highly suitable areas for *C. minutus*. This result supports the use of these two techniques to plan conservation actions. This pattern was not evident for *C. lami*, perhaps due to the restricted range of the species. Despite the current wide use of molecular markers to estimate genetic variation at different geographical scales and originating from different evolutionary processes [43], [44], it is still difficult to match these data with ecological parameters at broad scales. Moreover, despite many studies testing the relationship between genetic diversity and geographical populations, it is necessary to test alternative mechanisms, such as fragmentation and human effects on species distributions, by integrating genetic and demographic data in an ecological context.

## Supporting Information

Figure S1 **Input points given to the software (Maxent).**
(TIF)
