# Supplementary figures and images for: Genetic Pool Information Reflects Highly Suitable Areas: The Case of Two Parapatric Endangered Species of Tuco-tucos (Rodentia: Ctenomiydae)
Source: PLoS One. 2014 May 12;9(5):e97301. doi: 10.1371/journal.pone.0097301 (PMC4018344; doi:10.1371/journal.pone.0097301)

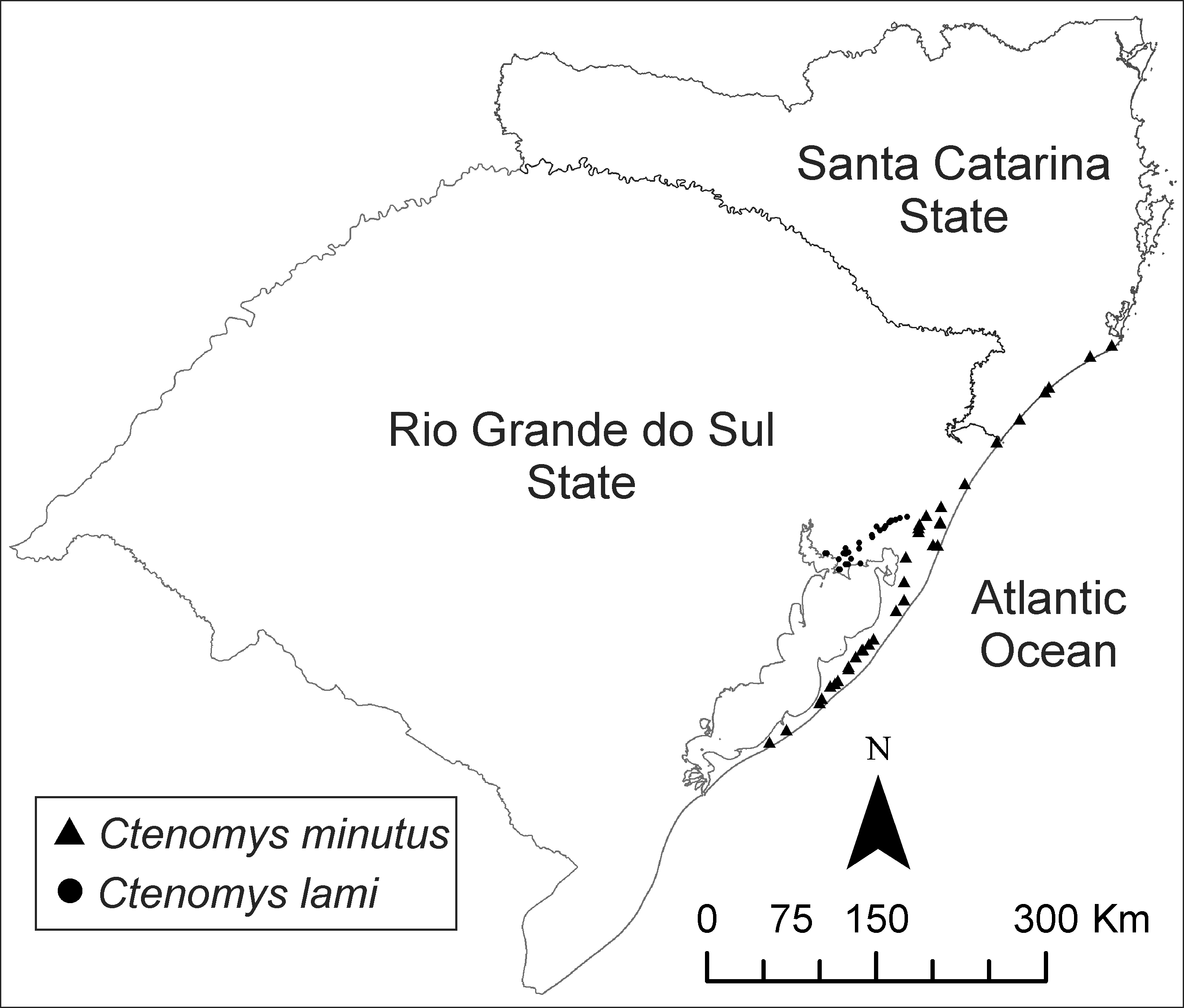

Supplement: Figure S1 — Input points given to the software (Maxent). (TIF) [file pone.0097301.s001.tif]
